# Beta-Sitosterol Promotes Milk Protein and Fat Syntheses-Related Genes in Bovine Mammary Epithelial Cells

**DOI:** 10.3390/ani11113238

**Published:** 2021-11-12

**Authors:** Xinlu Liu, Jinglin Shen, Jinxin Zong, Jiayi Liu, Yongcheng Jin

**Affiliations:** Department of Animal Science, College of Animal Science, Jilin University, 5333 Xi’an Road, Changchun 130062, China; liuxinlu629@163.com (X.L.); shenjinglinshen@aliyun.com (J.S.); zongjx19@163.com (J.Z.); loki980125@163.com (J.L.)

**Keywords:** bovine mammary epithelial cells, β-sitosterol, milk synthesis, mRNA and protein expression

## Abstract

**Simple Summary:**

The levels of milk fats and proteins are important indexes used to evaluate milk quality. Generally, feed additives are used to improve milk quality. This study aimed to investigate the effect of β-sitosterol on milk fat and protein gene expression in bovine mammary epithelial cells. β-sitosterol increased the β-casein levels in bovine mammary epithelial cells and promoted the expression of milk fat and protein synthesis-related genes, suggesting the use of β-sitosterol as a potential feed additive to improve milk quality in dairy cows.

**Abstract:**

β-sitosterol, a phytosterol with multiple biological activities, has been used in the pharmaceutical industry. However, there are only a few reports on the use of β-sitosterol in improving milk synthesis in dairy cows. This study aimed to investigate the effects of β-sitosterol on milk fat and protein syntheses in bovine mammary epithelial cells (MAC-T) and its regulatory mechanism. MAC-T cells were treated with different concentrations (0.01, 0.1, 1, 5, 10, 20, 30, or 40 μM) of β-sitosterol, and the expression levels of milk protein and fat synthesis-related genes and proteins were analyzed. β-sitosterol at 0.1, 1, and 10 μM concentrations promoted the mRNA and protein expression of β-casein. β-sitosterol (0.1, 1, 10 μM) increased the mRNA and protein expression levels of signal transducer activator of transcription 5 (STAT5), mammalian target of rapamycin (mTOR), and ribosomal protein S6 kinase beta-1 (S6K1) of the JAK2/STAT5 and mTOR signaling pathways. It also stimulated the milk fat synthesis-related factors, including sterol regulatory element-binding protein 1 (SREBP1), peroxisome proliferator-activated receptor-gamma (PPARγ), acetyl-CoA carboxylase (ACC), lipoprotein lipase (LPL), and stearyl CoA desaturase (SCD). β-sitosterol (0.1, 1, 10 μM) also significantly increased the expression of growth hormone/insulin-like growth factor-1 (GH/IGF-1) axis and hypoxia-inducible factor-1α (HIF-1α)-related genes. Notably, the compound inhibited the expression of the negative regulator, the suppressor of cytokine signaling 2 (SOCS2) at the two lower concentrations (0.1, 1 μM), but significantly promoted the expression at the highest concentration (30 μM). These results highlight the role of β-sitosterol at concentrations ranging from 0.1 to 10 μM in improving milk protein and fat syntheses, regulating milk quality. Therefore, β-sitosterol can be used as a potential feed additive to improve milk quality in dairy cows.

## 1. Introduction

Mammary epithelial cells synthesize caseins and whey proteins in milk [1]. Various signaling pathways, hormones, and nutrients regulate milk protein synthesis [2,3]. Janus kinase 2/signal transducer and activator of transcription 5 (JAK2/STAT5) and phosphoinositide 3-kinases/RAC-alpha serine/threonine-protein kinase/mammalian target of rapamycin (PI3K/AKT1/mTOR) pathways are the major pathways regulating milk protein synthesis in mammary epithelial cells [4]. Meanwhile, regulatory elements and enzymes such as sterol regulatory element-binding protein family (SREBPs), stearoyl-coenzyme A desaturase (SCD), and fatty acid synthase (FASN) regulate fatty acid synthesis by influencing de novo fat synthesis and absorption and fatty acid intake [5,6].

Several hormones and cytokines, such as prolactin, glucocorticoid, and insulin-like growth factor, influence the physiological function of mammary epithelial cells [7]. During mammary development and lactation, insulin is the main regulator of nutrient distribution in the lactating mammary glands. It also induces the expression of milk proteins by acting on the JAK2/STAT5 and PI3K/AKT/mTOR signaling pathway and enhances the expression of milk fat biosynthetic genes [8]. Insulin-like growth factor-1 (IGF-1) is an important upstream regulator of mTOR, affecting the proliferation and differentiation of mammary epithelial cells [9]. In rats and dairy cows, it enhances β-casein gene transcription in a concentration-dependent manner and significantly improves acetyl-CoA carboxylase (ACC) gene transcription, thereby enhancing milk protein yield and fat synthesis [10,11]. Researchers postulate that insulin-like growth factor-binding protein (IGFBP) and type 1 insulin-like growth factor receptor (IGF-1R) influence IGF-1 activity, which regulates growth and metabolism [12]. Additionally, growth hormone (GH) promotes proliferation, influences milk protein synthesis-related kinases and regulatory factors’ gene expression levels, and enhances the lactation ability of mammary epithelial cells [13]. Moreover, the suppressors of the cytokine signaling (SOCS) family terminate the effect of GH [14]. The SOCS family also maintains normal biological functions, such as cell proliferation and apoptosis, by negatively regulating signaling pathways, especially the JAK/STAT pathway.

β-sitosterol is a common phytosterol found in large quantities in plants, such as vegetables, fruits, and Chinese herbal medicine [15]. It has anti-inflammatory, antitumor, and lipid-lowering properties [16,17]. It also has estrogen-like effects that enhance the reproductive performance of the American mink [18]. Several researchers have studied and applied β-sitosterol in medicine and animal husbandry due to its multiple bioactive properties. However, the effects of β-sitosterol on milk synthesis in bovine mammary epithelial cells are poorly understood. We hypothesized that β-sitosterol might activate fatty acid synthesis through SREBP1-related genes and affect milk protein synthesis through JAK2/STAT5 and mTOR-related signaling pathways. Therefore, the present study analyzed the effects of β-sitosterol on the expression levels of the components of the GH/IGF-1 axis, JAK2/STAT5, and mTOR pathways and the genes related to milk fat synthesis, such as SREBP1 and PPARγ in vitro. The study aimed to investigate the effect of β-sitosterol on the expression of genes related to milk fat and protein syntheses using bovine mammary epithelial cells (MAC-T). The study provides evidence for the application of β-sitosterol in regulating milk protein and fat syntheses.

## 2. Materials and Methods

### 2.1. Cell Culture

Bovine mammary epithelial cells (MAC-T cells) were kindly provided by Professor Hong-Gu Lee (Konkuk University, Seoul, South Korea). These cells (passage number, fifty-five) were cultured in DMEM/high glucose medium (Hyclone, Logan, UT, USA) supplemented with 10% fetal bovine serum (Gibco, Gaithersburg, MD, USA), 1% penicillin-streptomycin (Hyclone, Logan, UT, USA), 5 μg/mL insulin (Sigma-Aldrich, St. Louis, MO, USA), and 1 μg/mL hydrocortisone (Sigma-Aldrich, St. Louis, MO, USA) in a 100 mm culture dish. The cells were subsequently cultured in the differentiation medium in a six-well culture dish for three days in a 37 °C cell incubator after attaining 100% confluence. The medium contained 5% fetal bovine serum, 5 μg/mL insulin, 1 μg/mL hydrocortisone, 5 μg/mL prolactin (Sigma-Aldrich, St. Louis, MO, USA), and 1% penicillin-streptomycin in DMEM/high glucose medium (Hyclone, Logan, UT, USA) and β-sitosterol (Chengdu Herbpurify, Chengdu, China) at various concentrations (0.01, 0.1, 1, 5, 10, 20, 30, or 40 μM). β-sitosterol was completely dissolved in ethanol (<0.1%) and stored in a refrigerator at 4 °C in the dark. Ethanol (<0.1%) was used as the solvent control. The induction medium was changed daily during differentiation. Three replicates were maintained per treatment.

### 2.2. Quantitative Real-Time PCR (qPCR) Analysis

Cells cultured in six-well plates were washed twice with phosphate-buffered saline (PBS) and scraped using a cell scraper with 1 mL of TRIzol reagent (Thermo Scientific, Waltham, MA, USA). The cell lysate was washed several times by passing through a pipette, transferring to a 1.5 mL Eppendorf tube, and storing at −80 °C until further analysis. Total RNA was subsequently extracted from the harvested MAC-T cells using TRIzol reagent following the manufacturer’s instructions for transcriptional analysis. The concentration and purity of the total RNA samples were measured using a NanoDrop 2000 spectrophotometer (Thermo Scientific, Waltham, MA, USA). The complementary DNA (cDNA) was synthesized from the RNA in a Life ECO Gene amplification instrument (BIOER, Hangzhou, China) using the HiFi Script cDNA Synthesis Kit (CWBIO, Beijing, China) according to the manufacturer’s instructions.

The qPCR was performed in a Stratagene Mx3005P system (Agilent Technologies, Santa Clara, CA, USA) using UltraSYBR mixture (CWBIO, Beijing, China). The qPCR thermal profile included an initial denaturation at 95 °C for 10 min followed by 40 cycles of amplification at 95 °C for 10 s, annealing at 60 °C for 30 s, and extension at 72 °C for 32 s. β-actin was used as the reference gene. Relative gene expression levels were quantified using the 2^−ΔΔCT^ method [19]. The sequences of primers used in the study are outlined in the Supplementary Material Table S1.

### 2.3. Western Blot Analysis

Cells cultured in six-well plates were washed twice with PBS and mixed with 1 mL of RIPA buffer (Sigma-Aldrich, St. Louis, MO, USA) containing 1 mM phenylmethanesulfonyl fluoride (PMSF; protease inhibitor; Sigma-Aldrich, St. Louis, MO, USA). The mixture was incubated for 30 min on ice, transferred to a 1.5 mL Eppendorf tube, and centrifuged at 12,000× *g* for 30 min at 4 °C to collect the supernatant for Western blotting. The protein concentration in each sample was measured using Bradford protein assay (Meilunbio, China). The proteins (10 µg) were separated by SDS-PAGE (10%, *w*/*v*) and transferred onto a nitrocellulose membrane (Millipore Corp, Billerica, MA, USA). The membrane was blocked for 3 h with 5% skim milk in Tris-buffered saline-Tween (TBST) buffer and incubated overnight at 4 °C with primary antibodies. The membrane was then incubated with an appropriate secondary antibody for 3 h at 4 °C after washing with TBST to wash off the excess primary antibodies. The membrane was then incubated with ECL Western blotting substrate (Meilunbio, China) and visualized using a chemiluminescence imaging system (Tanon, Shanghai, China). The protein bands were quantified using the GenoSens Gel analysis software (Gel Analysis Version 2.02, Shanghai, China). The level of each protein was normalized by comparing the signal with the β-actin signal on the same membrane. Details on antibodies are shown in the Supplementary Material Table S2.

### 2.4. Statistical Analysis

Data were analyzed using SPSS 17.0 (SPSS Inc., Chicago, IL, USA) and expressed as mean ± standard error (*n* = 3). The treatment effects were evaluated using one-way analysis of variance (ANOVA) followed by the Duncan multiple range test. Differences were considered statistically significant at *p* < 0.05.

## 3. Results

### 3.1. Effect of β-sitosterol on β-casein mRNA and Protein Expression in MAC-T Cells

**Figure 1:**
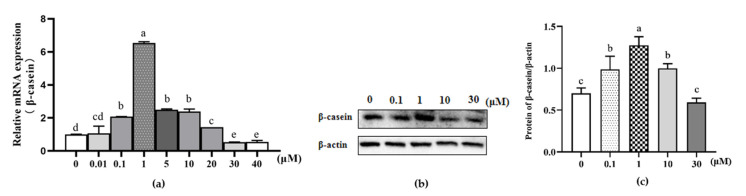
Effects of β-sitosterol on β-casein synthesis in MAC-T cells. (**a**) β-casein mRNA levels determined by qPCR. (**b**) β-casein protein levels detected by Western blotting. (**c**) The ratio of β-casein protein relative to β-actin control. Data are expressed as means ± standard deviation (*n* = 3). Different lower-case letters indicate significant differences compared to the control (*p* < 0.05).

The effect of β-sitosterol on the mRNA expression of β-casein in MAC-T cells was determined by qPCR (Figure 1). The mRNA expression of β-casein in MAC-T cells treated with β-sitosterol at low concentrations (0.1, 1, 5, 10 μM) was significantly upregulated compared to the control (*p* < 0.05, Figure 1a). In contrast, high concentrations of β-sitosterol (30, 40 μM) significantly inhibited the mRNA expression of β-casein compared to the control (*p* < 0.05; Figure 1a). Based on these data, β-sitosterol at 0.1, 1, 10, and 30 μM concentrations were selected for all subsequent experiments.

β-sitosterol at 0.1, 1, and 10 μM concentrations significantly increased the protein expression levels of β-casein (*p* < 0.05; Figure 1b,c), with 1 μM β-sitosterol exhibiting the highest protein expression of β-casein (*p* < 0.05; Figure 1b,c), consistent with the mRNA expression of β-casein. In contrast, the protein expression of β-casein remained unchanged in MAC-T cells treated with 30 μM β-sitosterol (Figure 1b,c). These results demonstrated that β-sitosterol stimulated the expression of β-casein.

### 3.2. Effect of β-sitosterol on mRNA Expression of Casein Synthesis-Related Genes and Phosphorylation of Casein Synthesis-Related Proteins in MAC-T Cells

**Figure 2:**
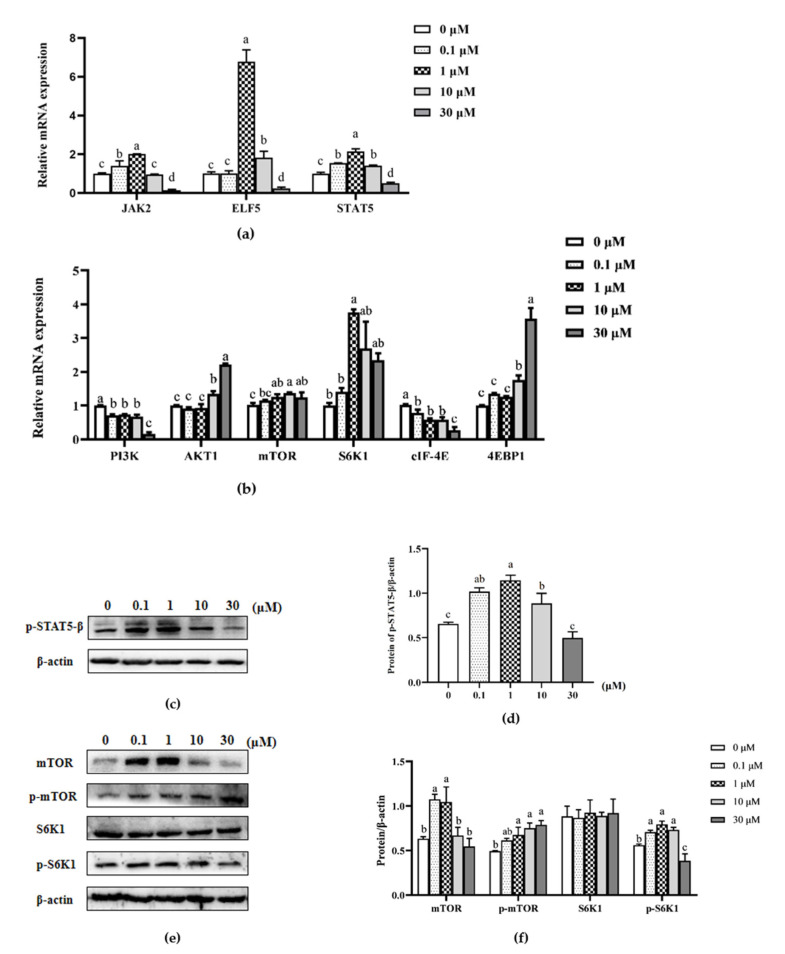
Effects of β-sitosterol on casein biosynthesis-related pathways in MAC-T cells. (**a**) mRNA expression of JAK2/STAT5 signaling pathway-related genes determined by qPCR. (**b**) mRNA expression of mTOR signaling pathway-related genes determined by qPCR. (**c**) Levels of p-STAT5-β protein detected by Western blotting. (**d**) The ratio of p-STAT5-β protein to β-actin control. (**e**) Levels of mTOR, p-mTOR, S6K1, and p-S6K1 proteins detected by Western blotting. (**f**) The ratio of mTOR, p-mTOR, S6K1, and p-S6K1 proteins to β-actin control. Data are mean ± SEM (*n* = 3). Different lower-case letters indicate significant differences compared to the control (*p* < 0.05).

qPCR and Western blotting were further used to analyze the mRNA expression of genes and the changes in proteins of the two major signaling pathways (JAK2/STAT5 and PI3K/AKT1/mTOR) related to milk protein synthesis in MAC-T cells under the action of β-sitosterol (Figure 2).

An analysis of the components of the JAK2/STAT5 pathway revealed that 1 μM β-sitosterol significantly increased the relative mRNA expression levels of JAK2, E74-like factor 5 (ELF5), and STAT5 (*p* < 0.05, Figure 2a), while 30 μM β-sitosterol significantly inhibited JAK2, STAT5, and ELF5 (*p* < 0.05; Figure 2a). In addition, 1 μM β-sitosterol significantly increased the p-STAT5-β protein levels (*p* < 0.05; Figure 2c,d), consistent with the mRNA levels, while 30 μM β-sitosterol did not affect the p-STAT5-β protein levels in MAC-T cells (Figure 2c,d). These results suggested that β-sitosterol enhanced JAK2/STAT5 signaling pathway expression.

Additionally, β-sitosterol inhibited the mRNA expression of PI3K (*p* < 0.05; Figure 2b), a component of the mTOR signaling pathway, at all concentrations. However, 10 and 30 μM β-sitosterol significantly increased the mRNA levels of downstream AKT1 and mTOR genes and the protein levels of p-mTOR (*p* < 0.05; Figure 2b,e,f). Among the downstream genes of the mTOR signaling pathway, the mRNA levels of the ribosomal protein S6 kinase beta-1 (S6K1) were upregulated in 1 μM β-sitosterol-treated cells (*p* < 0.05; Figure 2b). In the same line, the mRNA levels of eukaryotic translation initiation factor 4E-binding protein 1 (4EBP1) were higher in 10 and 30 μM β-sitosterol-treated cells (*p* < 0.05; Figure 2b). However, β-sitosterol inhibited the mRNA expression of eukaryotic translation initiation factor 4E (eIF-4E) at all concentrations (*p* < 0.05, Figure 2b). In addition, the S6K1 phosphate protein levels in cells treated with 0.1, 1, and 10 μM β-sitosterol were significantly higher than the control (*p* < 0.05; Figure 2e,f) but significantly lower in cells treated with 30 μM β-sitosterol (*p* < 0.05; Figure 2e,f). These results suggested that β-sitosterol promoted mTOR expression and enhanced the expression of downstream factor S6K1.

### 3.3. Effect of β-sitosterol on mRNA Expression of Fatty Acid Synthesis-Related Genes in MAC-T Cells

**Figure 3:**
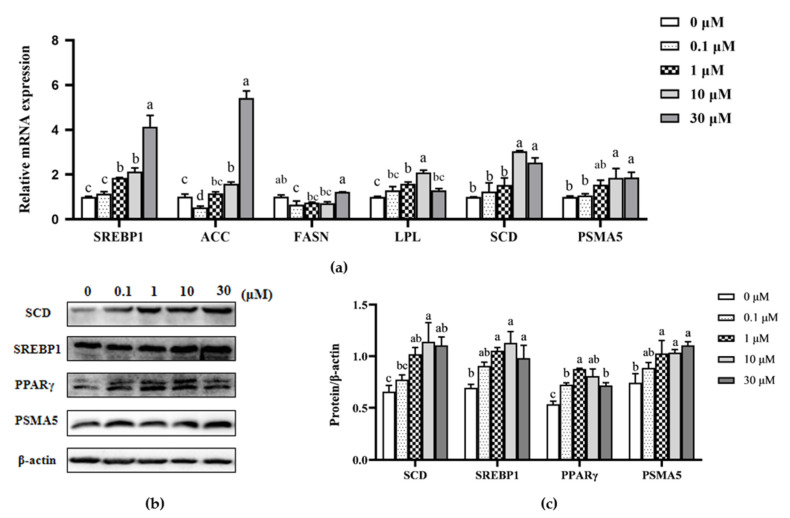
Effects of β-sitosterol on the expression levels of factors related to fatty acid synthesis in MAC-T cells. (**a**) mRNA expression of fatty acid synthesis-related genes determined by qPCR. (**b**) Protein levels of fatty acid synthesis-related factors detected by Western blotting. (**c**) The ratio of fatty acid synthesis-related factor proteins to β-actin control. Data are expressed as means ± SEM (*n* = 3). Different lower-case letters indicate significant differences compared to the control (*p* < 0.05).

The mRNA and protein levels of genes involved in de novo fatty acid synthesis and fatty acid uptake and decomposition regulation were further analyzed to determine the effect of β-sitosterol on milk fat synthesis in mammary epithelial cells. Treatment with 10 and 30 μM β-sitosterol significantly upregulated the mRNA levels of ACC (*p* < 0.05; Figure 3a). However, 0.1 μM β-sitosterol inhibited this expression (*p* < 0.05; Figure 3a). Meanwhile, the mRNA levels of FASN were suppressed in cells treated with 0.1 μM β-sitosterol (*p* < 0.05; Figure 3a). Notably, the mRNA levels of SCD and proteasome 20s subunit α5 (PSMA5) in cells treated with 10 and 30 μM β-sitosterol were significantly higher than the control (*p* < 0.05; Figure 3a), consistent with the protein expression levels (*p* < 0.05; Figure 3b). The mRNA expression levels of lipoprotein lipase (LPL) significantly increased in cells treated with 1 and 10 μM β-sitosterol (*p* < 0.05; Figure 3a). Similarly, the mRNA expression of SREPB1 in cells treated with 1, 10, and 30 μM β-sitosterol was significantly higher than in the control (*p* < 0.05; Figure 3a). It also promoted protein expression in all three concentrations (*p* < 0.05; Figure 3b,c). Moreover, the cells treated with β-sitosterol had an upregulated peroxisome proliferator-activated receptor-gamma (PPARγ) protein expression (*p* < 0.05; Figure 3b,c). These results suggested that β-sitosterol enhanced the expression of milk fat synthesis-related factors.

### 3.4. Effect of β-sitosterol on mRNA Expression of GH-IGF-1 Axis-Related Genes

**Figure 4:**
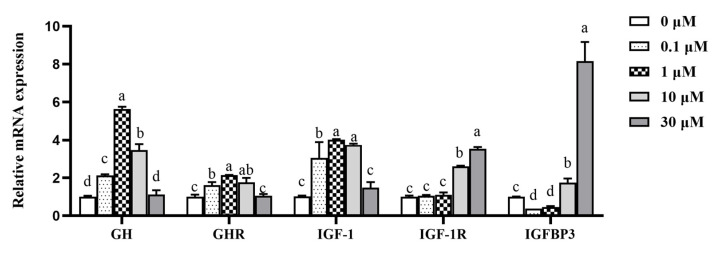
Effects of β-sitosterol on the expression levels of genes related to GH/IGF-1 axis in MAC-T cells. Data are expressed as means ± standard deviation (*n* = 3). Different lower-case letters indicate significant differences compared to the control (*p* < 0.05).

The expression levels of genes related to GH/IGF-1, an upstream regulatory pathway related to milk protein synthesis, were also analyzed. The GH and growth hormone receptor (GHR) genes in cells treated with 0.1, 1, and 10 μM β-sitosterol were significantly upregulated (*p* < 0.05; Figure 4), with 1 μM β-sitosterol exhibiting the maximum effect. Similarly, the IGF-1 mRNA expression in cells treated with 0.1, 1, and 10 μM β-sitosterol was significantly upregulated than the control (*p* < 0.05; Figure 4). β-sitosterol at concentrations of 10 and 30 μM also significantly increased the expression of the IGF-1R gene (*p* < 0.05; Figure 4). Similarly, 10 and 30 μM β-sitosterol significantly increased the expression of the IGFBP3 gene (*p* < 0.05; Figure 4), while 0.1 and 1 μM β-sitosterol significantly decreased the expression (*p* < 0.05; Figure 4).

### 3.5. Effects of β-sitosterol on the mRNA Expression of HIF-1α and Its Downstream Genes EPO and EPOR

**Figure 5:**
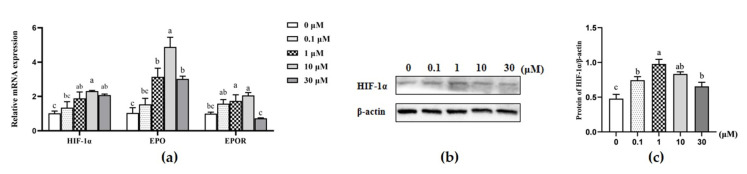
Effects of β-sitosterol on the expression levels of HIF-1α and related factors in MAC-T cells. (**a**) mRNA expression of HIF-1α and related factors determined by qPCR. (**b**) Levels of HIF-1α protein detected by Western blotting. (**c**) The ratio of HIF-1α protein to β-actin control. Data are expressed as means ± SEM (*n* = 3). Different lower-case letters indicate significant differences compared to the control (*p* < 0.05).

The expression of hypoxia-inducible factor-1α (HIF-1α) and its downstream genes erythropoietin (EPO) and erythropoietin receptor (EPOR) were analyzed based on the effects of β-sitosterol on the mTOR signaling pathway. The mRNA expression levels of HIF-1α and EPO were significantly upregulated in cells treated with 1, 10, and 30 μM β-sitosterol compared to the control (*p* < 0.05; Figure 5a), with 10 μM β-sitosterol exhibiting the maximum effect. In addition, 1 and 10 μM β-sitosterol upregulated the EPOR gene (*p* < 0.05; Figure 5a). Notably, the protein expression levels of HIF-1α were high in all β-sitosterol-treated groups (*p* < 0.05; Figure 5b,c).

### 3.6. Effect of β-sitosterol on the mRNA and Protein Expression of SOCS2 and SOCS3

**Figure 6:**
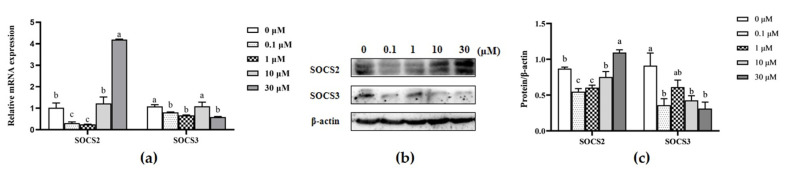
Effects of β-sitosterol on the expression levels of SOCS in MAC-T cells. (**a**) mRNA expression of SOCS2 and SOCS3 genes determined by qPCR. (**b**) Levels of SOCS2 and SOCS3 proteins detected by Western blotting. (**c**) The ratio of SOCS2 and SOCS3 proteins to β-actin control. Data are mean ± SEM (*n* = 3). Different lower-case letters indicate significant differences compared to the control (*p* < 0.05).

The negative feedback regulatory elements of the JAK2/STAT5 pathway, such as SOCS2 and SOCS3, were analyzed based on the effect of β-sitosterol on GH/IGF-I axis, JAK2/STAT5, and mTOR signaling pathways. The expression of the SOCS2 gene was significantly inhibited in the 0.1 and 1 μM β-sitosterol-treated groups than in controls (*p* < 0.05; Figure 6a), but significantly upregulated in MAC-T cells treated with 30 μM β-sitosterol (*p* < 0.05, Figure 6a). Meanwhile, the expression of the SOCS3 gene was significantly inhibited in the 0.1, 1, and 30 μM β-sitosterol-treated groups than in controls (*p* < 0.05; Figure 6a). Western blot analysis revealed that the changes in the protein expression levels were consistent with those of the mRNA expression levels (Figure 6b,c).

### 3.7. Correlation Heat Map Analysis

**Figure 7:**
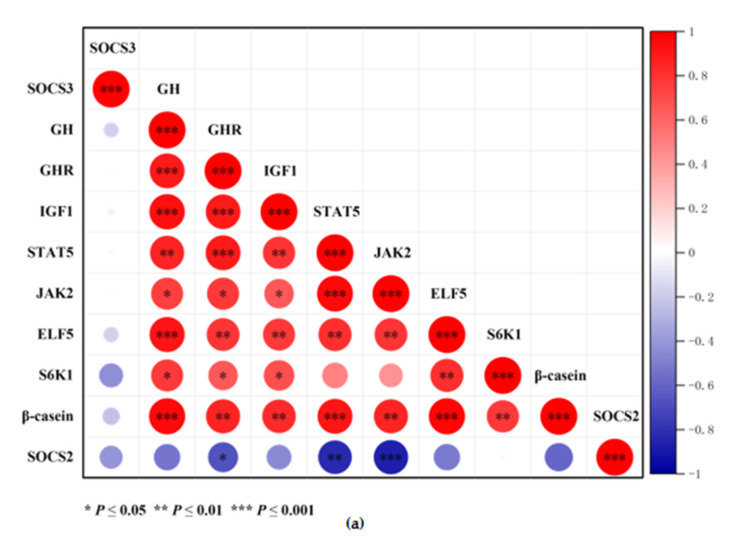

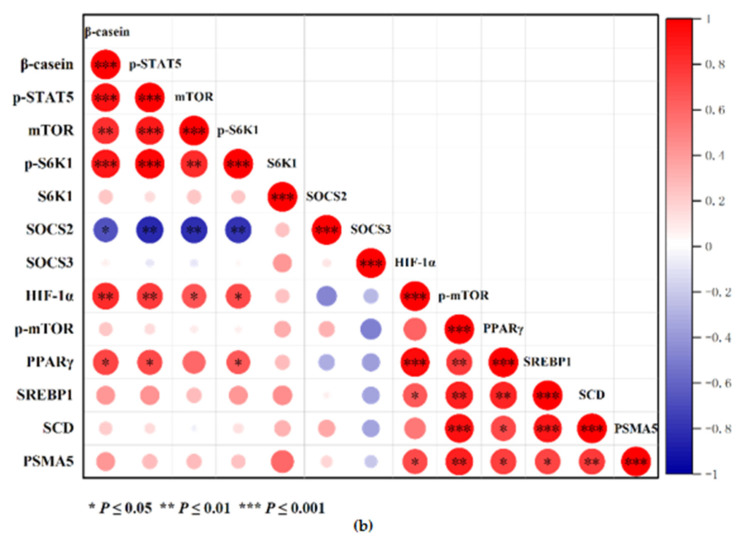
Correlation and thermographic analysis of mRNA and protein expression. (**a**) Correlation heat map of gene expression. Blue indicates a negative correlation, and red indicates a positive correlation; the circles’ size and color show the correlations between the genes. (**b**) Correlation heat map of protein expression. Symbols *, **, and *** indicate significant correlations between genes or proteins at *p* ≤ 0.05, *p* ≤ 0.01, and *p* ≤ 0.001, respectively.

The correlation between gene and protein expression levels was analyzed, and heat maps were generated (Figure 7) using the Origin software (Origin Pro 2021, Version 9.8.0.200) and the Correlation Plot App (Origin Version 9.75). The genetic correlation analysis (Figure 7a) revealed a positive correlation between GH and GHR, IGF-1, β-casein (*p* ≤ 0.001), and STAT5 (*p* ≤ 0.01). IGF-1 was positively correlated with S6K1 and β-casein (*p* ≤ 0.05). However, SOCS2 was negatively correlated with GHR, STAT5, and JAK2 (*p* ≤ 0.05). Meanwhile, the protein correlation analysis (Figure 7b) revealed a positive correlation of β-casein with p-STAT5 and p-S6K1 (*p* ≤ 0.001). p-mTOR was positively correlated with SREBP1, PPARγ, PSMA5, and SCD (*p* ≤ 0.01). In addition, HIF-1α was positively correlated with β-casein, p-STAT5, SREBP1, and PPARγ (*p* ≤ 0.05). However, SOCS2 was negatively correlated with β-casein, p-STAT5, p-mTOR, and p-S6K1 (*p* ≤ 0.05).

### 3.8. Schematic Diagram of the Pathways Involved in β-sitosterol-Mediated Regulation of Milk Fat and Protein Synthesis

**Figure 8:**
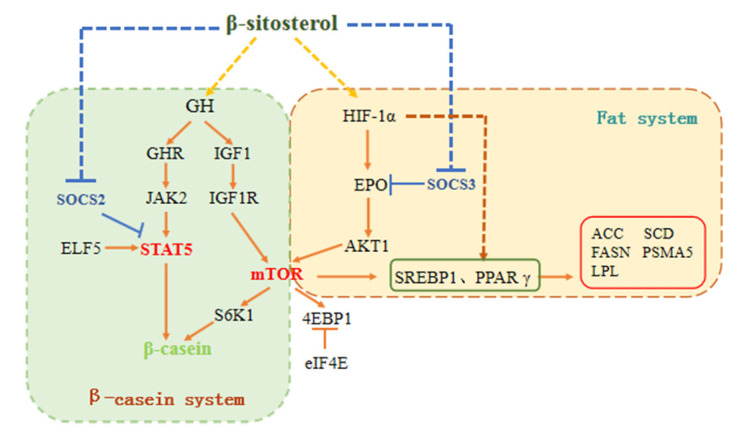
Schematic diagram showing the regulation of milk fat and protein syntheses by β-sitosterol in bovine mammary epithelial cells. “↓” indicates a positive effect; “⊥” indicates an inhibitory effect. The solid lines represent signaling pathways confirmed by previous studies, whereas the dotted lines represent the putative pathways based on the results of this study. GH = growth hormone; IGF-1 = insulin-like growth factor-I; GHR = growth hormone receptor; IGF-1R = type 1 insulin-like growth factor receptor; JAK2 = janus kinase 2; STAT5 = signal transducer activator of transcription 5; ELF5 = E74-like factor 5; mTOR = mammalian target of rapamycin; S6K1 = ribosomal protein S6 kinase beta-1; 4EBP1 = eukaryotic translation initiation factor 4E binding protein 1; eIF-4E = eukaryotic initiation factor 4E; SREBP1 = sterol regulatory element-binding protein 1; PPARγ = peroxisome proliferator-activated receptor γ; ACC = Acetyl-CoA carboxylase; FASN = fatty acid synthase; LPL = lipoprotein lipase; SCD = stearyl CoA desaturase; PSMA5 = proteasome 20 s subunit α5; HIF-1α = hypoxia-inducible factor-1α; AKT1 = RAC-alpha serine/threonine-protein kinase; EPO = erythropoietin; SOCS = suppressors of cytokine signaling.

The experimental results and correlation analysis suggested that β-sitosterol regulated the GH/IGF-1 axis, affected SOCS expression, and regulated milk fat and protein synthesis signaling pathways in bovine mammary epithelial cells because of its estrogen-like properties. β-sitosterol enhanced the expression of the JAK2/STAT5 signaling pathway by activating the GH axis and inhibiting SOCS2, subsequently promoting β-casein synthesis. It promoted mTOR expression by activating IGF-1 and HIF-1α/EPO and upregulating the mTOR downstream factor S6K1, thereby affecting β-casein synthesis. In addition, β-sitosterol enhanced the expression of SREBP1 and PPARγ by activating HIF-1α and promoted the expression of milk fat synthesis-related factors (Figure 8).

## 4. Discussion

### 4.1. β-sitosterol Affects JAK2/STAT5 and mTOR by Regulating the GH/IGF-1 Axis, Thus Promoting Milk Protein Synthesis

GH, a peptide hormone with important physiological functions, promotes fat decomposition and protein synthesis, thus regulating growth and development [20]. Studies postulate that it has lactation-promoting effects and improves milk yield, milk component yield, and feed conversion efficiency of dairy cows [21,22,23,24,25,26]. GH either directly acts on GHR or stimulates IGF-1 synthesis [27]. It binds to the transmembrane receptor (GHR) and subsequently undergoes a conformational change to induce the JAK/STAT signal transduction pathway by recruiting and activating JAK2 [28,29,30,31]. The phosphorylated STAT5 dimer protein interacts with the promoter of milk protein genes, thereby promoting the transcriptional expression of the casein gene [32,33]. In this study, low concentrations of β-sitosterol (0.1, 1, and 10 μM) increased the mRNA expression levels of GH, GHR, JAK2, STAT5, and β-casein and increased the protein levels of p-STAT5 and β-casein. These findings strongly suggested that β-sitosterol plays a significant role in regulating JAK2/STAT5 and casein expression through GH.

Studies postulate that GH stimulates IGF-1 synthesis and secretion [34,35]. This study detected a similar effect, revealing a positive correlation between the mRNA expression of IGF-1 and GH and GHR. Studies also postulate that IGF-1 exhibits some lactation-promoting effects. Its content in the blood was positively correlated with lactation in dairy cows [36]. Meanwhile, IGF-1 regulates the mTOR signaling pathway and functions after binding to the receptor (IGF-1R) [9]. The binding protein (IGFBP) and IGF-1R also influence IGF-1 activity [11]. Chicharro et al. reported that lower IGFBP3 contents had a stronger IGF-1 effect on the tissues [37]. In this study, low concentrations of β-sitosterol promoted mRNA expression of GH, GHR, and IGF-1 but inhibited mRNA expression of IGFBP3. Additionally, β-sitosterol inhibited mRNA expression of PI3K, upregulating the mRNA levels of AKT1 and mTOR and mTOR phosphorylation. Notably, β-sitosterol resulted in the maximum mRNA and protein expression levels of downstream factors S6K1 at a concentration of 1 μM. These results collectively suggest that the effects of β-sitosterol on the mTOR signaling pathway are not regulated by PI3K but by IGF-1/IGF-1R [9].

Furthermore, the heat map analysis revealed that the GH, GHR, and IGF-1 mRNA levels were positively correlated with JAK2, ELF5, STAT5, and S6K1, consistent with previous reports [38]. Generally, estrogen increases the GH content by stimulating the neuroendocrine growth axis and regulating IGF-1 generation and IGF-1 binding protein utilization, thus influencing mammary gland growth and development [39]. Collectively, these findings suggest that β-sitosterol regulates the JAK2/STAT5 and mTOR signaling pathways through the GH/IGF-1 axis to influence casein synthesis.

### 4.2. β-sitosterol Activates HIF-1α/EPO and Promotes Milk Protein and Fat Synthesis

Insulin (INS) is an important hormone that regulates the nutrient distribution and milk fat and protein syntheses in lactating mammary glands [40]. β-sitosterol attenuates insulin resistance by reducing insulin receptor substrate-1 (IRS-1) serine phosphorylation and activating downstream signaling molecules in rats [41]. Studies postulate that insulin stimulates HIF-1α [42], a nuclear transcription factor activated under hypoxic conditions, in a concentration-dependent manner [43]. Notably, it regulates a series of hypoxia signal pathways to enhance the expression of erythropoietin (EPO) and its receptor (erythropoietin receptor, EPOR) genes [44], thereby promoting the proliferation and differentiation of mammalian erythroid progenitor cells. Studies also postulate that EPO has anti-inflammatory, antiapoptotic, neuroprotective, and other non-erythropoietic effects [45]. Its function depends on the EPO-EPOR signal transduction mechanism [46,47], which induces downstream factors, such as the PI3K/AKT pathway, for further activation. Wu et al. found that PC12 cells treated with recombinant EPO had a continuous activation of AKT [46]. EPO also activates mTOR and enhances its activity during hypoxia-reoxygenation stress in hippocampal-derived neurons [48]. In this study, β-sitosterol upregulated the expression of HIF-1α, EPO, EPOR, AKT1, and mTOR to a certain extent. HIF-1α was positively correlated with mTOR, suggesting that β-sitosterol regulates mTOR through HIF-1α/EPO.

In mammary tissues, the mTOR pathway affects the elements guiding milk fat syntheses, such as SREBPs and PPARγ, thereby regulating fatty acid synthesis [4,5,6,49]. SREBPs and PPARγ are important transcriptional regulators in the fatty acid synthesis gene network that directly regulates genes, such as ACC and SCD, which affect the transport and synthesis of milk fat [50,51]. Various factors, such as hormones (insulin, growth hormone), affect SREBP1, PPARγ, and ACC [27,52]. In addition, ubiquitination-related components play an important role in fat synthesis [53]. Proteasome 20 s subunit α5 (PSMA5) is a degradable protein that promotes the assembly of 20 s proteasomes and affects ubiquitination [54]. Jin et al. reported the role of PSMA5 in the endogenous synthesis of conjugated linoleic acid (CLA) in the goat mammary gland and MAC-T cells [55]. In this study, 10 and 30 μM β-sitosterol upregulated SCD, SREBP1, PPARγ, and PSMA5 proteins. These findings suggested that it can be used as a potential regulatory nutrient for the endogenous synthesis of fatty acids, such as conjugated linoleic acid, in the mammary epithelial cells to improve milk quality. The heat map analysis revealed that p-mTOR was positively correlated with SREBP1, PPARγ, and SCD. These results collectively indicate that β-sitosterol regulates the expression of SREBP1 and PPARγ through mTOR to increase the expression of milk fat synthesis-related factors.

The heat map analysis also revealed that HIF-1α was positively correlated with PPARγ and SREBP1. Researchers postulate a correlation between HIF-1α and fatty acid metabolism; HIF-1α activates SREBP-1 and stimulates FASN [56,57]. The present study revealed a positive correlation between HIF-1α, β-casein, and p-STAT5, consistent with the role of HIF-1α in promoting STAT5 phosphorylation in mammary epithelial cells [58]. In summary, β-sitosterol acts on the HIF-1α pathway and affects the downstream genes, regulating milk fat and protein syntheses.

### 4.3. β-sitosterol Inhibits SOCS Expression and Promotes the Expression of Milk Protein and Fat Synthesis-Related Factors

SOCS is a class of protein factors that negatively regulate cytokines. They are involved in many physiological and pathological processes, including growth, metabolism, bone formation, oncogenesis, and immunity [59]. Studies postulate that SOCS inhibits a variety of signaling pathways. SOCS2 inhibits the GH/IGF-1 axis and negatively regulates growth and development after birth [60,61], whereas SOCS3 inhibits the EPO signaling pathways [62]. Moreover, previous studies report a close association between the signaling pathways and the lactation regulatory factors [63,64]. This study analyzed the expression and protein levels of SOCS2 and SOCS3 genes to decipher their involvement in the expression of milk protein and fat synthesis-related factors. The expression and protein levels of the SOCS2 gene were inhibited by 0.1 and 1 μM β-sitosterol, while 30 μM β-sitosterol increased the expression. These results were consistent with the effect on the STAT5 and GH/IGF-1 axis in MAC-T cells. In contrast, 10 and 30 μM β-sitosterol significantly decreased the protein expression levels of SOCS3. These findings were consistent with the regulation of HIF-1α/EPO in MAC-T cells. These observations collectively indicate that β-sitosterol regulates the GH/IGF-1 axis and HIF-1α/EPO signaling pathway to influence STAT5 and mTOR expression by affecting the expression of the SOCS family. This study’s findings elucidate the mechanism underlying the beneficial role of β-sitosterol at the cellular and molecular levels. The study also highlights the potential of β-sitosterol in promoting the production of milk components in dairy cattle.

## 5. Conclusions

β-sitosterol concentrations between 0.1 and 10 μM activate the JAK2/STAT5 and mTOR signaling pathways, thus affecting the milk fat synthesis-related genes and proteins, such as SREBP1 and PPARγ, and promoting β-casein synthesis in bovine mammary epithelial cells. The study’s findings indicate that β-sitosterol is a potential feed additive for dairy cows to improve milk quality. However, further in vivo feeding experiments should be conducted to verify its effectiveness.

## Data Availability

The data presented in this study are available on request from the corresponding author.

## Supplementary Materials

The following are available online at https://www.mdpi.com/article/10.3390/ani11113238/s1, Table S1: Bos taurus (cattle) primers used for qPCR, Table S2: Antibodies used for Western Blot.
