# Supplementary material for: Beta-Sitosterol Promotes Milk Protein and Fat Syntheses-Related Genes in Bovine Mammary Epithelial Cells
Source: Animals (Basel). 2021 Nov 12;11(11):3238. doi: 10.3390/ani11113238 (PMC8614283; doi:10.3390/ani11113238)
Supplement: Supplementary file 1 [file animals-11-03238-s001.zip › animals-1385288-supplementary.pdf]

animals

MDPI

Supplementary material

Beta-Sitosterol Promotes Milk Protein and Fat Syntheses-Related Genes in Bovine Mammary Epithelial Cells

XinLu Liu †, JingLin Shen †, JinXin Zong, JiaYi Liu and YongCheng Jin *

Department of Animal Science, College of Animal Science, Jilin University, 5333 Xi'an Road, Changchun 130062, China; liuxinlu629@163.com (X.L.); shenjinglinshen@aliyun.com (J.S.); zongjx19@163.com (J.Z.); loki980125@163.com (J.L.)

* Correspondence: ycjin@jlu.edu.cn

† These authors contributed equally to this work.

Citation: Liu, X.; Shen, J.; Zong, J.; Liu, J.; Jin, Y. Beta-Sitosterol Promotes Milk Protein and Fat Syntheses-Related Genes in Bovine Mammary Epithelial Cells. *Animals* **2021**, *11*, 3238. https://doi.org/10.3390/ani11113238

Academic Editor: Donata Marletta

Received: 13 September 2021
Accepted: 10 November 2021
Published: 12 November 2021

Publisher's Note: MDPI stays neutral with regard to jurisdictional claims in published maps and institutional affiliations.

Copyright: © 2021 by the authors. Licensee MDPI, Basel, Switzerland. This article is an open access article distributed under the terms and conditions of the Creative Commons Attribution (CC BY) license (http://creativecommons.org/licenses/by/4.0/).

Table S1. Bos taurus (cattle) primers used for qPCR.

Gene Name [1]	Accession Number	Primers Sequence	Product Size,bp
β-actin	NM_173979.3	F: 5′-CCCTGGAGAAGAGCTACGAG-3′<br>R:5′-GTAGTTTCGTGAATGCCGCAG-3′	130
β-casein	XM_015471671.2	F: 5′-GGCTATGGCTCCTAAGCACA-3′<br>R:5′-GTCAGGCTCTGCCTTTCAGT-3′	78
JAK2	XM_005209981.4	F: 5′-CAAGACCAGATGGATGCCCAG-3′<br>R:5′-ACTCGAACTGCTAGGTCTCTGA-3′	103
STAT5	NM_174617.4	F: 5′-GAGAACACCCGCAATGATTAC-3′<br>R:5′-TCACCGACTCTGCTCCACG-3′	151
ELF5	NM_001024569.1	F: 5′-CATCCGCTCACAAGGTTACTC-3′<br>R:5′-CTCGCACAAATTCCCATAGAT-3′	170
PI3K	NM_001206047.1	F: 5′-GTCTGGACCTTCGGATGCTAC-3′<br>R:5′-TAAACTCCTCAATGGCTCGGT-3′	213
AKT1	NM_173986.2	F: 5′-GCACAAGCGAGGTGAGTACAT-3′<br>R:5′-GCCACGGAGAAGTTGTTGAG-3′	138
mTOR	XM_002694043.6	F: 5′-CGAAGAACCAATTATACCCGC-3′<br>R:5′-CATAGCAACCTCAAAGCAGTCC-3′	153
S6K1	NM_205816.1	F: 5′-AATGCTGCTTCTCGTCTTGGA-3′<br>R:5′-CAGTTCTTCCCAGTTAATATGTCT-3′	90
4EBP1	NM_001077893	F: 5′-TCACTAGCCCTACAGGCGAT-3′<br>R:5′-AAACTGTGACTCTTCACCGC-3′	101
eIF4E	NM_174310.3	F: 5′-CCCGCCTACAGAAGAAGAGA-3′<br>R:5′-CAGTATCAAACTTAGAGATCAATCG-3′	164
ACC	NM_174224.2	F: 5′-GGAGACAAACAGGGACCATTAC-3′<br>R:5′-GTGGAAGGAATGCTTGGGAG-3′	187
FASN	NM_001012669.1	F: 5′-GACCTGGGAGGAGTGTAAGC-3′<br>R:5′-GCGATAGCGTCCATGAAGTA-3′	198
SCD	NM_173959.4	F: 5′-CCACGTTCTTCATTGATTGC-3′<br>R:5′-CAGCCACTCTTGTAGCTTTCC-3′	121
LPL	NM_001075120.1	F: 5′-TCACTTCAACCACAGCAGCA-3′<br>R:5′-GATGACGTTGGAGTCCGGTT-3′	127
SREBP1	NM_001113302.1	F: 5′-CGCTCTTCCATCAATGACA-3′<br>R:5′-TTCAGCGATTTGCTTTTGTG-3′	188
PSMA5	NM_001015566.1	F: 5′-CATGAGTGGGCTAATTGCTG-3′<br>R:5′-AGCCTGAGTCACACTCTCCA-3′	115
GH1	NM_180996.1	F: 5′-GCAGATCCTCAAGCAGACCT-3′<br>R:5′-CAGGAGAGCAGACCGTAGTT-3′	87
GHR	NM_176608.1	F: 5′-AGACCACTTCTCATTGGTGGA-3′<br>R:5′-ATGTCGCTTACCTGGGCATA-3′	110
IGF-1	NM_001077828.1	F: 5′-CCATCACATCCTCCTCGCAT-3′<br>R:5′-ATAAAAGCCCCTGTCTCCGC-3′	143
IGF-1R	NM_001244612.1	F: 5′-CACGAGTGGAGAAATCTGCG-3′<br>R:5′-ATGTGGAGGTAGCCCTCGAT-3′	102
IGFBP3	NM_174556.1	F: 5′-AGCGTGAGACAGAATACGGG-3′<br>R:5′-AGCCCTTCTTGTCGCAGTTG-3′	120
HIF-1α	NM_174339.3	F: 5′-TTCCATCTCCTCCCCACGTA-3′<br>R:5′-AGGCTGTCCGACTTCCAGTA-3′	81
EPO	NM_173909.2	F: 5′-TCTCCCTTCCAGATGCAACC-3′<br>R:5′-CTTTCCCCGCAGGAAATTGG-3′	104
EPOR	NM_001205601.1	F: 5′-CTCGTCCTCGTGCTCATTCT-3′	117

		R:5′-GCCTTCAAACTCGCTCTCAG-3′	
SOCS2	NM_177523.2	F: 5′-GGGAACTCAGTCACACAGGTTGG-3′<br>R:5′-TGTTAGTAGGTAGTCTGAATGCGAAC-3′	128
SOCS3	NM_174466.2	F: 5′-AGAAGATCCCTCTGGTGTTGAGC-3′<br>R:5′-GTGACTTTCTCGTAGGAGTCCAGG-3′	109

[1] *JAK2* = Janus kinase 2; *STAT5* = Signal transducer activator of transcription 5; *mTOR* = Mammalian target of rapamycin; *S6K1* = Ribosomal protein S6 kinase beta-1; *SREBP1* = Sterol regulatory element-binding protein 1; *PPARγ* = Peroxisome proliferator-activated receptor γ; *ACC* = Acetyl-CoA carboxylase; *FASN* = Fatty acid synthase; *LPL* = Lipoprotein lipase; *SCD* = Stearyl CoA desaturase; *GH* = Growth hormone; *IGF-1* = Insulin-like growth factor-I; *HIF-1α* = Hypoxia-inducible factor-1α; *SOCS* = Suppressors of cytokine signaling; *PI3K* = Phosphoinositide 3-kinase; *AKT1* = RAC-alpha serine/threonine-protein kinase; *IGFBPs* = Insulin-like growth factor binding protein; *IGF-1R* = Type 1 insulin-like growth factor receptor; *ELF5* = E74-like factor 5; *4EBP1* = Eukaryotic translation initiation factor 4E binding protein 1; *eIF-4E* = Eukaryotic initiation factor 4E; *PSMA5* = Proteasome 20s subunit α5; *GHR* = Growth hormone receptor; *EPO* = Erythropoietin; *EPOR* = Erythropoietin receptor.

Materials and Methods:

HiFi Script cDNA Synthesis Kit (CWBIO, Beijing, China) according to the manufacturer's instructions. The reaction was carried out in a volume of 20 μL containing 4 μL of dNTP mix, 2 μL of primer mix, 4 μL of 5 × RT buffer, 2 μL of DTT, 1 μL of HiFi Script, and 1μg of the RNA template, and the cDNA was synthesized by incubating the reaction mixture at 42℃ for 15 min followed by 85℃ for 5 min.

Table S2. Antibodies used for Western Blot.

Antibodies name	Diluted multiples	Accession Number	Reagent company
rabbit anti-β-actin polyclonal antibody	1:2000	bs-0061R	Bioss
rabbit anti-β-casein polyclonal antibody	1:2000	bs-10032R	Bioss
rabbit anti- phospho-STAT5-β polyclonal antibody	1:2000	bs-5703R	Bioss
rabbit anti-mTOR polyclonal antibody	1:2000	bs-1992R	Bioss
rabbit anti-phospho-mTOR polyclonal antibody	1:2000	bs-3492R	Bioss
rabbit anti-S6K1 polyclonal antibody	1:2000	bs-6370R	Bioss
rabbit anti- phospho-S6K1 polyclonal antibody	1:2000	bs-5668R	Bioss
rabbit anti-SCD polyclonal antibody	1:2000	bs-3787R	Bioss
mouse anti-PSMA5 polyclonal antibody	1:2000	bs-51520R	Bioss
rabbit anti-SREBP1 polyclonal antibody	1:2000	bs-1402R	Bioss
rabbit anti-PPARγ polyclonal antibody	1:2000	bs-0530R	Bioss
rabbit anti-HIF-1α polyclonal antibody	1:2000	bs-0737R	Bioss
rabbit anti-SOCS2 polyclonal antibody	1:2000	bs-1896R	Bioss
rabbit anti-SOCS3 polyclonal antibody	1:2000	bs-24250R	Bioss
goat anti-rabbit IgG antibody	1:4000	ab-6721R	abcam
rabbit anti-mouse IgG antibody	1:4000	ab-6728R	abcam
